# Quantification of Movement in Stroke Patients under Free Living Conditions Using Wearable Sensors: A Systematic Review

**DOI:** 10.3390/s22031050

**Published:** 2022-01-28

**Authors:** Mariano Bernaldo de Quirós, E.H. Douma, Inge van den Akker-Scheek, Claudine J. C. Lamoth, Natasha M. Maurits

**Affiliations:** 1 Department of Neurology, University Medical Center Groningen, University of Groningen, 9700 RB Groningen, The Netherlands; m.bernaldo.de.quiros@umcg.nl; 2 Department of Human Movement Sciences, University Medical Center Groningen, University of Groningen, 9700 RB Groningen, The Netherlands; helmydouma@hotmail.com (E.H.D.); c.j.c.lamoth@umcg.nl (C.J.C.L.); 3 Department of Orthopedics, University Medical Center Groningen, University of Groningen, 9700 RB Groningen, The Netherlands; i.scheek@umcg.nl

**Keywords:** stroke, activities of daily living, continuous monitoring, wearables, movement quantification

## Abstract

Stroke is a main cause of long-term disability worldwide, placing a large burden on individuals and health care systems. Wearable technology can potentially objectively assess and monitor patients outside clinical environments, enabling a more detailed evaluation of their impairment and allowing individualization of rehabilitation therapies. The aim of this review is to provide an overview of setups used in literature to measure movement of stroke patients under free living conditions using wearable sensors, and to evaluate the relation between such sensor-based outcomes and the level of functioning as assessed by existing clinical evaluation methods. After a systematic search we included 32 articles, totaling 1076 stroke patients from acute to chronic phases and 236 healthy controls. We summarized the results by type and location of sensors, and by sensor-based outcome measures and their relation with existing clinical evaluation tools. We conclude that sensor-based measures of movement provide additional information in relation to clinical evaluation tools assessing motor functioning and both are needed to gain better insight in patient behavior and recovery. However, there is a strong need for standardization and consensus, regarding clinical assessments, but also regarding the use of specific algorithms and metrics for unsupervised measurements during daily life.

## 1. Introduction

Stroke is classically characterized as a neurological deficit attributed to an acute focal injury of the central nervous system by a vascular cause and is a major cause of disability and death worldwide [1]. Around 80% of stroke victims survive [2], but their quality of life can be severely impacted in both physical and physical-emotional domains [3]. The physical effects of a stroke in the brain mainly manifest on the contralateral side of the body and can be extremely persistent: research shows that 3–6 months after stroke, 55–75% of stroke survivors still experience problems in functioning of the affected body side [4].

Motor functioning of stroke patients is typically assessed in the controlled environment of a clinic, hospital or research laboratory, usually by asking the patient to perform standard clinical assessment tests which include repetitive tasks or isolated movements. However, this functional assessment is not representative of individual free-living behavior [5]. Because daily life functioning is severely affected by stroke [3], monitoring patients during their activities of daily living (ADL) could provide more valid information about patients’ functioning in their home environment. This knowledge enhances evaluation of the effects of rehabilitation interventions, which could help to improve the interventions and quality of care and thus eventually stroke patients’ quality of life. 

Recent technological developments in wearable technology have led to a steady increase in the number of studies monitoring movements related to activities of daily living of stroke patients. These wearable systems benefit from a high acceptance rate and simplicity and can be used independently from a base station, which makes them easy to use outside controlled environments. However, most studies that use wearable measurement systems in stroke patients are using them to measure repetition of a task or routine in clinical or simulated ADL conditions in well controlled settings. Measurements in these controlled conditions may result in performance bias and are thus not representative of the actual patients’ functioning in their home environment [6]. Additionally, those measurement systems used in simulated ADL environments might not be reliable for examining daily life functioning in free-living environments: movements in the latter less organized environment are self-initiated, usually task oriented, less predictable and have a higher variation [7].

Lately, an increasing number of studies have tried to gain better insight into the real-life behavior of patients by evaluating them in their free-living environment [8,9,10,11]. Using wearable sensors quantitative aspects of movement, such as the amount of activity, the number of steps or repetitions or the intensity of movement can be determined. However, for functioning in daily life qualitative aspects of movement are also important, since they provide information about how movements are performed, and insight into those movement aspects that need to be tailored during rehabilitation. Since continuous monitoring of stroke patients’ functioning in daily life has inherent challenges that are not present in the clinic or lab, a clear overview of the possibilities for capturing quantitative and qualitative aspects of movement through sensor-based assessment would be of great interest. Furthermore, an overview of relations between such sensor-based measures and existing clinical evaluation tools would provide insight in the validity and added value of continuous movement monitoring in stroke patients. 

A few prior studies executed reviews that are related to this topic. The study of Noorkõiv et al. [12] is most comparable in scope to ours: with the aim of assessing the additional clinical value of accelerometry after stroke they selected eight studies that investigated upper extremity activity after stroke, in free-living environments and discussed correlations between accelerometry and clinical measures. However, many developments in this area of research have happened in the last decade. Another interesting review for our purposes is the one by Johansson et al. [13] who studied the use of wearable sensors for clinical applications in stroke, as well as epilepsy and Parkinson’s disease. Their aim was to synthesize knowledge from quantitative and qualitative clinical studies, executed in laboratory, hospital as well free living environments, including studies of movement as well as physical activity. They included 24 studies in stroke but did not provide an overview of measurement set-ups. Gebruers et al. [14] systematically reviewed clinimetric properties and clinical applicability of different accelerometry-based measurement techniques in stroke patients. With that aim in mind, they also discussed correlations between accelerometry and common stroke scales when reported in included studies. They did not specifically focus on activity in free living conditions, however, nor did they discuss or summarize the measurement setups used. Fini et al. [15] described how physical activity is monitored following stroke, summarising methods and devices used across the stroke pathway and documenting their psychometric properties. They did not study quantification of movement nor did they focus on free living conditions, however. The most recent related review [16] focused on how wearable technologies have been used over the past decade to assess gait and mobility, but not other types of movement, in stroke patients. They did not focus on free living conditions, either. In summary, there is no recent overview of how quantitative and qualitative aspects of movement are captured through sensor-based assessment in stroke patients, during free-living conditions. Furthermore, a recent overview of relations between such sensor-based measures and existing clinical evaluation tools in this context is also missing.

The aim of this review is therefore: (1) to provide an overview of setups used in literature to measure the quantitative and qualitative aspects of movements of stroke patients under free living conditions using wearable sensors, and (2) to evaluate the relation between the sensor-based outcomes that are obtained from moving in a free living environment and the level of functioning as assessed by existing clinical evaluation methods.

## 2. Materials and Methods

This review was performed according to the preferred reporting items for systematic reviews and meta-analysis statement (PRISMA) [17] and registered in the international prospective register of systematic reviews (PROSPERO registration ID: CRD42020207226).

### 2.1. Search Method

A literature search was conducted on the 30 December 2021, using the PubMed, Scopus and Web of Science databases.

The search term was composed by four extensive parts separated by the AND operators:

(1) Terms for stroke
(2) Terms for movement and motor symptoms
(3) Terms for wearable sensors and devices
(4) Terms for activities of daily living and continuous monitoring

This resulted in the following detailed search term (in PubMed format):

(“Stroke”[Mesh] OR Cerebrovascular Accident*[tiab] OR Stroke*[tiab] OR CVA[tiab])
AND
(“Movement”[Mesh] OR “Motor Disorders”[Mesh] OR Move*[tiab] OR Motor Symptom*[tiab] OR Motor Disorder*[tiab])
AND
(Accelerometer*[tiab] OR “IMU”[tiab] OR Inertial Unit*[tiab] OR Gyroscope*[tiab] OR “Electrical Equipment and Supplies”[Mesh] OR Sensor*[tiab] OR Wearable*[tiab] OR Tracker*[tiab] OR Emg[tiab] OR Electromyograph*[tiab] OR Pressure Sens*[tiab] OR Strain Gauges Based Sens*[tiab] OR Strain Sens*[tiab] OR Strain Gauge*[tiab])
AND
(“Activities of Daily Living”[Mesh] OR Activities of Daily Living[tiab] OR ADL[tiab] OR Daily life*[tiab] OR “Continuous Monitoring”[tiab] OR “Remote Monitoring”[tiab] OR “Monitoring, Physiologic”[Mesh] OR “Monitoring, Ambulatory”[Mesh] OR Home-Based[tiab] OR “Environment”[Mesh] OR “Environment*”[tiab] OR “Communal*”[tiab] OR “Commune*”[tiab] OR “Community*”[tiab] OR “Communities”[tiab] OR “Free-Living”[tiab] OR “Free Living”[tiab] OR “Long Term”[tiab] OR “Real World”[tiab])

After combining the results of these searches, three steps were taken to identify eligible studies. First, one author (MB) removed duplicates. Second, two authors (MB, HD) screened the remaining papers independently by titles and abstracts, using the inclusion/exclusion criteria specified below. Third, remaining full-text versions were assessed independently by two authors (MB, HD). Any disagreements in the results were resolved in a consensus meeting with a third assessor (CL).

### 2.2. Eligibility Criteria

Included studies involved patients after a stroke episode, used wearable sensor technology for movement assessment and quantifying patient movement. Articles in other languages than English as well as abstracts, case reports, reviews, study protocols, non-original research or theses were excluded. Furthermore, as we aim to only include studies under free living conditions, by which we mean that participants carried out their normal routine at the clinic or at home without any behavioral constraints or instructions, studies were excluded if they used only standardized assessments or tests, if participants performed a list of scripted activities, or if they used non-passive setups, such as robotic assistance, electrical stimulation or feedback systems. Studies were also excluded if the main topic was activity recognition or measurement of the amount of physical activity, e.g., by energy expenditure. Finally, studies taking place during the hyper-acute phase of stroke (less than one day after stroke) were also excluded on the rationale that patients in such an early phase—in most cases bedridden and clinically monitored—can hardly perform any kind of activities of daily living.

### 2.3. Assessment of Methodological Quality

Each of the selected articles was assessed on methodological quality by two authors (MB, HD) using the tool developed by Downs and Black [18] (Supplementary Materials). For items 3–5, 10, 19, 25 and 27, we added an explanation to the table in Supplementary Materials on how the items were interpreted in the context of our review. In case of inconsistencies regarding the scores on the items, disagreements were resolved by consensus or by discussion with a third assessor (CL). The study quality was classified as high (score ≥14, 75% of the maximum score of 19), moderate (9 ≤ score < 14, 50–74% of the maximum score) or low (score < 9, 50% of the maximum score). Low-quality studies were excluded from further data extraction and synthesis.

### 2.4. Data Extraction and Synthesis

The data were extracted by two authors (MB, HD) using pre-formatted forms that included authors and year of publication, experimental design, sensor technology and placement, measurement task, population, outcomes extracted (clinical measures, sensor-based measures) and results. 

Note that the aim of the review was not to provide a meta-analysis of results; due to the wide variety in the design of studies and outcome measures used it was not possible to pool outcome measures together. Therefore, a narrative synthesis was created, covering the main descriptive themes from the selected articles relevant for our research questions, such as characteristics of the sensor setups and relations between sensor-based movement characteristics and the patient’s clinical state. The latter were expressed in correlation strength. 

## 3. Results

**Figure 1:**
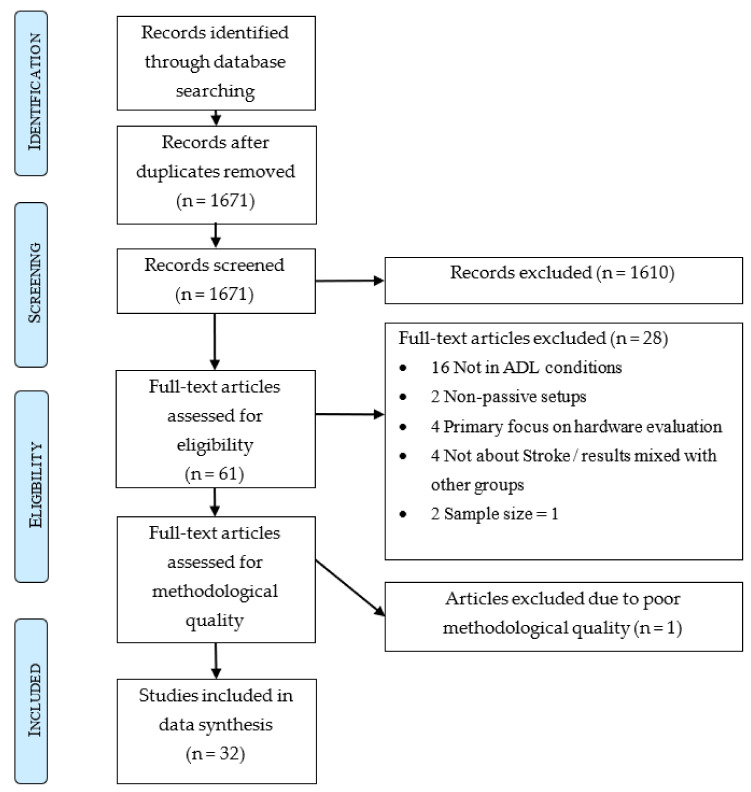
PRISMA graph.

The database search yielded 2561 studies. After removal of duplicates, 1671 remained. Application of the eligibility criteria excluded 1610 articles during title and abstract screening and 28 articles during full-text screening. The remaining 33 articles were assessed for methodological quality. One study was excluded due to poor methodological quality, scoring only four points (Supplementary Materials). Out of the remaining 32 studies that were included for data synthesis, 29 (91%) were of high quality [5,8,9,10,11,19,20,21,22,23,24,25,26,27,28,29,30,31,32,33,34,35,36,37,38,39,40,41,42] and three (9%) of moderate quality [43,44,45]. This inclusion process is described in Figure 1. A summary of all included studies is presented in Appendix A in Table A1 (continuous recording in the clinic or during rehabilitation) and Table A2 (continuous recording during ADL).

### 3.1. Study Design, Sample Size and Participant Characteristics

All 32 included studies were observational, of which 17 were cross-sectional and 15 were prospective cohort studies. Sixteen studies assessed activities in the hospital or rehabilitation center (Appendix A; Table A1), and sixteen studies activities of daily living in the home environment (Appendix A; Table A2). Eleven studies compared results between stroke patients and healthy controls and five studies had their stroke patients divided into two groups for comparison between fallers and not-fallers [11], walking patients and wheelchair users [5], inpatients and outpatients [25] or controls and patients following Constraint Induced Movement Therapy (CIMT) [26,27] or other interventions [41]. 

In total 1076 stroke patients were included in the 32 studies, ranging from four to 169 stroke patients per study. Ten studies included data of in total 236 healthy controls in their analysis [5,8,20,23,29,33,35,36,40,44,45]. The reported average age of the stroke participants was 61 years while healthy participants were usually younger with a reported average of 55.8 years.

Reported Time After Stroke (TAS) was highly different between studies: five studies took place during the acute phase of stroke [33,38,40,42,43]—less than a week, as defined by Bernhardt et al. [46]—another eight included patients during the early sub-acute phase [5,10,21,22,25,30,34,39] and eleven focused on chronic stroke patients [8,9,11,20,26,27,28,29,36,41,44]. The remaining articles included patients which were in different phases after stroke onset: acute and early sub-acute [23,24], early-subacute and late subacute [19], late sub-acute and chronic [35] or early, late and chronic phase [31,32,37,45]. See Appendix A for details.

### 3.2. Protocol

All studies consisted of at least one hour of continuous measurement. In 16 of the included studies measurements were performed in pure ecological conditions at the home of the participants (Appendix A; Table A2). In the other 16 studies participants were measured or started being measured in the hospital or rehabilitation center (Appendix A, Table A1). The clinical motor assessment instruments differed between studies, however, the free-living part of their protocols was in essence the same: it started with the instrumentation of the participant by the researcher, after which they could move freely during the length of the assessment. In five studies participants were measured in both clinical and home environments (Appendix A, Table A1 and Table A2). The time patients were measured ranged from 2 to 168 h. In the home environment the mean measurement duration was 73 ± 66.5 h while it was 54.5 ± 69.5 h in the hospital/rehabilitation environment. The longest measurements were 24 h a day for at least 7 consecutive days in the home and hospital environments [11,39,43]. In total 7936 h of healthy participant and 100,815 h of patient data were gathered, the latter divided into 72,463 h in home environments and 28,352 h at hospitals and rehabilitation centers.

### 3.3. Sensor Placement and Technology

**Figure 2:**
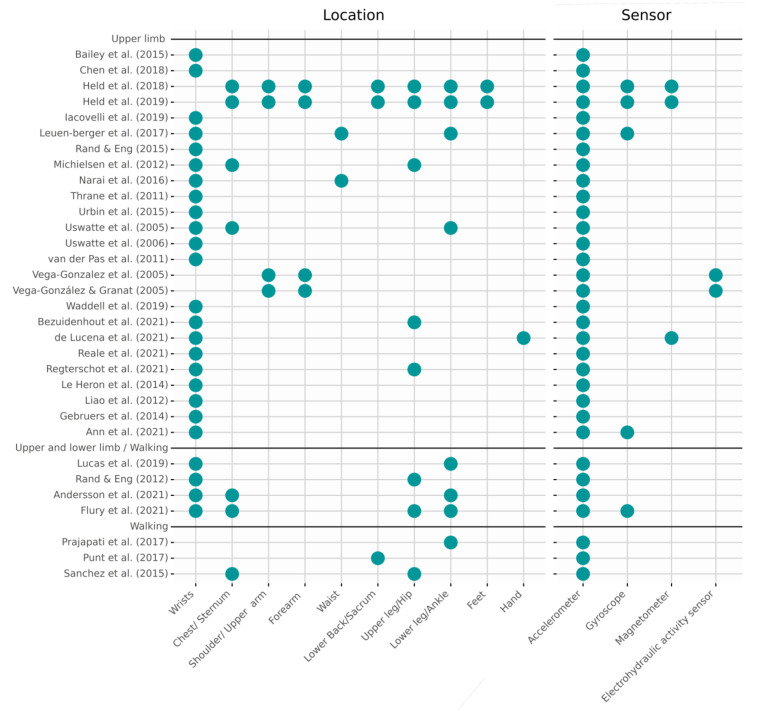
Sensor technology and locations. Overview of the locations of the sensors and type of sensors used in the included studies, separated by the focus of the study. Note that several upper limb studies include sensors on other parts of the body, but these are not used in the analysis or are used for secondary purposes, such as activity recognition or development of a kinematic model of the body [5,8,9,10,11,19,20,21,22,23,24,25,26,27,28,29,30,31,32,33,34,35,36,37,38,39,40,41,42,43,44,45].

Accelerometers and activity monitors—with embedded accelerometer sensors—were used in all studies (Figure 2). Five studies used inertial measurement units (IMUs) [19,31,32,34,45] that combine tri-axial accelerometers, gyroscopes and magnetometers, although three of them [19,34,45] did not include the data from the magnetometer in their analysis. The other two studies [31,32] used IMUs as part of a full body motion measuring system. Two studies of the same research group [29,44] used an activity monitor combined with an electrohydraulic activity sensor that, by means of a fluid-filled tube laid over the arm from shoulder to wrist, was able to measure the elevation of the arm with respect to the body. Finally, de Lucena et al. [36] used a setup made from an accelerometer, four magnetometers and a magnetic ring to measure the amount of hand activity.

Most of the studies—26 of 32—focused their research exclusively on upper limb functioning and placed the sensors on the wrists or forearms, while twelve of these studies also placed sensors on other parts of the body [10,19,20,26,29,31,32,35,36,39,43,44]. Except for the studies [29,44], in which the (electrohydraulic) sensors were placed along the arm, from shoulder to wrist, and [36] in which a magnetic ring was used as part of the setup, the information of the additional sensors was used to derive parameters for full body kinematic models [31,32], activity recognition or gait detection [10,19,20,26,35,39]. Four studies combined upper and lower body data [5,34,37,43], and three focused on walking only, in particular on the amount, number of walking bouts and gait characteristics [11,21,23]. While the wrists were clearly the preferred location of upper body studies, gait and lower body performance were evaluated by using single or a combination of sensors on the sternum, lower back, hips, thighs and ankles [11,21,23].

### 3.4. Movement Measures Derived from Sensors

In total 110 different variables were reported that were calculated from the sensor signals, to examine quantitative (amount of movement) and qualitative (symmetry, variability, kinematics) aspects of upper limb activity and gait performance. 

#### 3.4.1. Upper Limb Activity-Related Movement Measures 

Most of the upper limb activity related movement measures were based on the magnitude of the 3D accelerometer vector $\sqrt{x^{2}+y^{2}+z^{2}}$. Several studies [5,8,9,10,19,20,22,24,25,28,30,35,36,39,40,41,42,43] integrated this value over a time window -ranging from a few seconds [8] to several hours [10]- to quantify the amount and intensity of upper limb movement. Studies that reported their findings using similar values, like the signal magnitude area [34], or ‘counts’ are also included in this category. Counts represent filtered and summed acceleration signals, created by the software of manufacturers of several commercially available activity trackers. While counts also employ the magnitude of the acceleration, the algorithms used in creating them are not always available, which limits comparability between activity trackers. A simplification of the movement magnitude is the use time, which is calculated by applying a threshold to the amount of movement over a period of time, to determine what is movement and what is noise. Use time is usually calculated using the norm of the acceleration, except in the study by Flury et al. [37], that uses an algorithm based on the information from triaxial gyroscopes. From these two values—magnitude of movement and use time—measures of quantitative as well as qualitative aspects of movement can be calculated.

Additionally, six studies [19,29,31,32,44,45], analyzed the quality of movement by calculating kinematic measures from IMUs and electrohydraulic sensors, and one study [36] focused on the number of hand movements using a custom setup and algorithm.

#### 3.4.2. Measures of Quantitative Aspects of Upper Limb Movement

*Unilateral and bilateral magnitude.* Unilateral magnitude refers to the amount of movement of either the affected upper limb (AUL) or the unaffected upper limb (UUL) of stroke patients. When comparing unilateral magnitude of stroke patients with controls the AUL is usually compared to the non-dominant upper limb (NDUL) of controls, and the UUL to the dominant upper limb (DUL) of controls. In eleven studies [5,8,9,10,19,20,22,28,34,39,43] these measures were used. Bilateral magnitude refers to the amount of simultaneous upper limb movement (AUL + UUL), calculated per time window [8,28,30].

*Unilateral, bilateral and total use time*. Six studies [8,19,20,24,27,37] reported about the total time spent over a magnitude threshold for individual AUL and UUL (DUL and NDUL in controls) movements. *Bilateral use time* was reported by four studies [8,20,29,44] and is defined as the total time of performing simultaneous upper limb movements. Finally, by adding up the unilateral use time and simultaneous bilateral use time *Total use time* is obtained [8,29].

#### 3.4.3. Measures of Qualitative Aspects of Upper Limb Movement

Symmetry of arm movement, usually expressed as a ratio, is one of the main qualitative outcomes evaluated in the literature. *Magnitude ratio* was reported in ten studies [8,10,19,24,25,28,30,34,35,39] and refers to the magnitude of AUL acceleration relative to the magnitude of the UUL acceleration (AUL/UUL), per time window. It represents the relative contribution of each arm to the activity, and is an indicator of symmetry in use intensity. Similarly, the *Use Ratio* [25,26,27,29,30,37,44] refers to the use time of the AUL relative to the use time of the UUL and is an indicator of symmetry of upper arm use. For both magnitude and use ratio values near 1 indicate more symmetric movement.

Variability of arm movement as reported in three studies [25,33,38] is also used to asses the quality of upper limb movement. *Acceleration variability* is usually calculated as the standard deviation of the acceleration norm σ, per 1 min epoch [33] or over the entire monitoring period [25]. Additionally, the norm of the standard deviation of the acceleration vector components $\sqrt{\sigma_{x}^{2}+\sigma_{y}^{2}+\sigma_{z}^{2}}$, is used as another measure of acceleration variability that may be more sensitive to rotation movements [33,38]. *Variation Ratio*, both a variability and a symmetry outcome, is calculated over the total recording period [25], or for every 24 h [33] as the magnitude of AUL acceleration variability relative to the magnitude of UUL acceleration variability. We include in this category the score proposed by Le Heron et al. [40], because it is both a measure of variability and symmetry between AUL and UUL.

Finally, arm kinematic measures provide several metrics of movement quality. *Average* *joint range of motion* was reported in one study [31], describing the degree of motion of elbow and shoulder. Additionally, several reaching related measures of movement were identified. *Reaching area* [31] is defined as the encircled trajectory of the hand position relative to the pelvis in the horizontal plane and describes the degree of motion of elbow and shoulder. The maximum distance of the hand relative to the pelvis defines *reaching distance* [31]. *Reaching counts* refers to the number of times the hand showed a displacement of more than 10 cm away from the preferred hand position [31,32] and *reaching ratio* is calculated as the ratio of reaching counts of the AUL relative to the UUL [31]. *Distribution of forearm elevation*, reported in three studies [19,29,44], is based on the distribution of the forearm elevation relative to the body over time. It is reported as a probability distribution [19] or as movement time spent in separated vertical regions relative to the body [29,44]. *Gross arm movement time* is the duration, during the recording period, of movements in which the sum of a change of forearm orientation in yaw and elevation is more than 30° within a time period of 2 s, but only if the movement occurs within a range of forearm elevation between −30° and +30° [19,45].

#### 3.4.4. Hand Movement Related Measures

Only one study focused on the study of hand movements during ADL [36]. This study proposes the quantitative measure ‘HAND counts’ measuring the amount of movement of the fingers with respect to the wrist, where the sensors are located. 

#### 3.4.5. Lower Body and Gait Related Measures

One study [34] studied lower limb activity using sensor measures equivalent to the ones used for the upper limb, namely unilateral magnitude of the affected lower limb (ALL) and unaffected lower limb (ULL) and magnitude ratio (ALL/ULL). Six included studies [5,11,21,23,37,43] reported gait related measures obtained from walking bouts during ADL. The extracted gait related measures were both quantitative and qualitative and are widely used throughout gait studies. Quantitative gait measures were *duration of walking*, *number of steps* and *number of walking bouts* [5,21,23,37] while the measures of qualitative aspects of movement were *gait symmetry, walking speed*, *stride time*, *stride (step) regularity*, *standard deviation of accelerations*, *harmonic ratio*, *index of harmonicity*, *frequency*, *amplitude* and *width of the dominant power peak*, *local divergence exponent* and *step-time ratio* [5,11,21,23,43].

#### 3.4.6. General Measures of Quantitative Aspects of Movement

By applying human activity recognition (HAR) algorithms, four of the included studies evaluated the duration of patient activities during the day, providing quantitative measures of movement. The main measures of this type were percentage of activity time during the day, i.e., how long patients engage in dynamic activities or passive activities expressed as a percentage of the day [20,23,35,37]. 

#### 3.4.7. Comparison of Movement Measures with Clinical Assessment Tools

**Figure 3:**
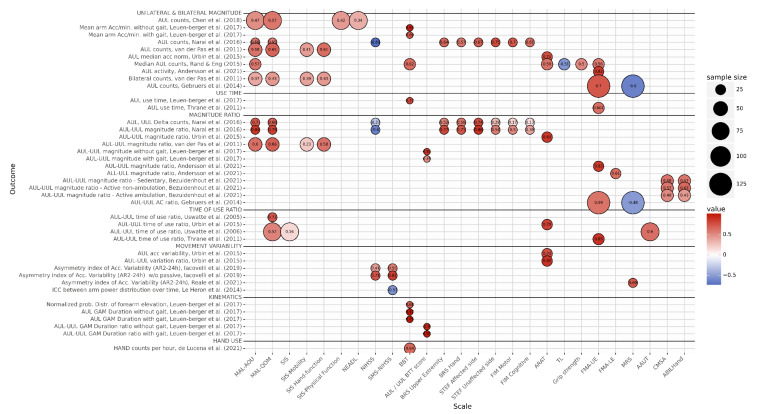
Correlation Between outcomes and Clinical Scales. Outcomes have been grouped by unilateral and bilateral magnitude, use time, magnitude ratio, time of use ratio, movement variability and kinematic outcomes. Color represents correlation value with the clinical scales, with hot colors expressing positive correlation and cold colors negative correlation. Size of the circles represents the size of the sample of the related study. The name of the outcomes remains as close as possible as in the original study. List of abbreviations: AC = activity counts, Acc = Acceleration, AR2-24 h = Asymmetry Rate Index (see ref.), ALL = Affected Lower Limb, AUL = Affected Upper Limb, GAM = Gross Arm Movement, ULL = Unaffected Lower Limb, UUL = Unaffected Upper Limb. Clinical Scales: AAUT = Actual Amount of Use Test, ARAT = Action Research Arm Test, BBT = Box and Blocks Test, BRS(-UE, -H) = Brunnstrom Recovery Stage (Upper Extremity, Hand), CMSA = Chedoke McMaster Stroke Assessment Scale, FIM (-M, -C) = Functional Independence Measure (Motor, Cognitive), FMA(-UE)(-LE) = Fugl-Meyer Assessment (Upper Extremity)(Lower Extremity), MAL(-AOU, -QOM) = Motor Activity Log (Amount of Activity, Quality of Movement), MRS = Modified Rankin Scale, NEADL = Nottingham Extended Activities of Daily Living, (SMS-)NIHSS = (Supplementary Motor Scale) National Institute of Health Stroke scale, SIS = Stroke Impact Scale, STEF = Simple Test for Evaluating Hand Function, TL = Thumb Localization Test [9,10,19,22,24,25,26,27,28,33,34,35,36,38,40,42].

Sixteen studies compared sensor-based movement measures with clinical assessment tools [9,10,19,22,24,25,26,27,28,33,34,35,36,38,40,42]. All studies used Pearson or Spearman correlation tests to determine the relationships between sensor-based measures and clinical assessment scale scores, assuming a significance level of α = 0.05. Correlation values were interpreted as follows: 0.00–0.30 = weak; 0.31–0.50 = low; 0.51–0.70 = moderate; 0.71–0.90 = strong; 0.91–1.00 = very strong. An overview of the correlations between the different sensor-based movement measures and clinical assessment tools, in relation to sample size and grouped by unilateral and bilateral magnitude, use time, magnitude ratio, time of use ratio, movement variability, kinematic and hand movement outcomes is presented in Figure 3. Here, the results are discussed per clinical assessment tool.

The Motor Activity Log (MAL) is a semi-structured questionnaire that assesses the patient’s perception of how much the affected arm is used and the quality of movement of its use during normal daily activities at home. These two aspects of upper limb functioning are captured by the MAL’s two subscales: Amount of Use (AOU) and Quality of Movement (QOM). The MAL uses a 5-point Likert scale, where a higher score reflects a better ability to use the affected arm [47]. The MAL-AOU was assessed in four studies [9,10,22,28] and the MAL-QOM in five studies [9,10,26,27,28]. Unilateral upper limb magnitude had a low-to-moderate correlation with both MAL-AOU (r = 0.37–0.58) and MAL-QOM (r = 0.43–0.65) [9,10,22,28]. The correlation between MAL-AOU and MAL-QOM and magnitude ratio ranged from strong [10] (r = 0.84, r = 0.79, respectively) to moderate (r = 0.6, r = 0.66 respectively) [28]. Use ratio had a moderate-to-strong correlation with MAL-QOM (r = 0.52–0.71) [26,27]. These findings imply a positive relation between a more balanced use of UUL and AUL and both a better perception of use of the AUL and a higher quality of movement of that limb, as captured by the MAL.

The Stroke Impairment Scale (SIS) for clinical assessment, is a self-reported index of overall physical activity. Participants rate their ability to function in their daily environment on a 5-point Likert scale, where a higher score reflects the experience of fewer difficulties [48]. One of the articles used the total SIS score [27], one used the Physical Function subscale [9] and one used the Hand Function and Mobility subscales [28]. Unilateral and bilateral magnitude of upper limb function had a low to moderate correlation with the Physical Function (unilateral: r = 0.42) [9], Hand Function (unilateral: r = 0.61, bilateral: 0.43) and Mobility (unilateral: r = 0.41, bilateral: r = 0.39) subscale scores [28]. Magnitude ratio had a low correlation with the Mobility subscale score (r = 0.23) and a moderate correlation with the Hand Function subscale score (r = 0.58) [28]. However, the use ratio was only weakly correlated (r = 0.16) with the total SIS score [27], indicating that overall self-reported ability of physical functioning is low to moderately correlated to the aforementioned sensor-based metrics. 

The National Institute of Health Stroke Scale (NIHSS) is an impairment scale consisting of 15 items and has been widely used in clinical trials and as initial assessment tool after stroke, with a higher score reflecting more severe stroke symptoms [48]. The NIHSS was used in two studies [10,33], and the Supplementary Motor Scale of the NIHSS (SMS-NIHSS) was assessed by two studies [33,40]. In this supplementary scale, motor function of the shoulder, wrist, hip and ankle is assessed, while the NIHSS only includes proximal motor function. The correlation of unilateral magnitude and magnitude ratio with NIHSS was moderately negative (r = −0.69, r = −0.60, respectively), implying that more severe stroke symptoms are characterized by low absolute AUL use and low AUL use compared to UUL use [10]. Le Heron et al. (2014) found a moderate correlation with their index—similar to variation ratio—and SMS-NIHSS (r = −0.53) and Iacovelli et al. (2019) found that the correlation between the asymmetry index of the acceleration variability and both NIHSS and SMS-NIHSS was moderate (r = 0.41, r = 0.51) but strong (r = 0.71, r = 0.81, respectively) for the second index proposed, which is more sensitive to rotational movements. These results imply that more severe stroke symptoms are characterized by a larger difference in variability in acceleration between upper limbs, especially during rotational movements.

The Box and Blocks Test (BBT) can be considered a fast screening tool for assessing gross manual dexterity, with a higher score reflecting better gross manual dexterity. The BBT provides information about the speed of performance but qualitative information on movement performance is not fully captured [49]. The correlation between several metrics, computed with and without periods of walking, and the BBT ranged from moderate to very strong [19]. Unilateral magnitude in- and excluding walking was moderately and very strongly correlated with the BBT score (r = 0.69, r = 0.93, respectively). Similarly, the correlation of the magnitude ratio AUL/UUL with the BTT increased from r = 0.49 to r = 0.84 when excluding walking implying that removal of walking periods from the data positively affects the correlation between these metrics and the BBT score. BBT score had a strong correlation with unilateral use time including walking (r = 0.77), a moderate correlation with distribution of forearm elevation probability excluding walking (r = 0.68), a moderate correlation with the amount of hand use [36] and a strong correlation with gross movement duration (r ≥ 0.90) [19]. Furthermore, Rand & Eng (2015) also found a moderate correlation between BBT and unilateral magnitude of the affected arm (r = 0.62). These results indicate a moderate to strong correlation of sensor-based measures and the BBT score. It should be noted, however, that one of these studies only had 10 stroke participants [19], making these correlations less reliable. 

The Action Research Arm Test (ARAT) is a test for upper limb functioning that consists of 19 items which are divided into 4 subscales: grasp, grip, pinch and gross movement. A higher score on the ARAT reflects better upper limb functioning [49]. Unilateral AUL magnitude was moderately correlated with the ARAT score in [22], while Urbin et al. [25] found a strong correlation between the ARAT and five sensor-based metrics: AUL median acceleration magnitude (r = 0.75), use ratio (r = 0.79), magnitude ratio (r = 0.83), acceleration variability (r = 0.73) and variation ratio (r = 0.85). The strong correlation between these variability and ratio metrics and the ARAT score implies that better function is characterized by a more dynamic and symmetrical use of AUL and UUL. 

Other clinical scales that were considered assessed functioning after rehabilitation or during ADL, activity level or real-world arm use based on spontaneous behavior. The Fugl-Meyer upper extremity motor assessment scale (FMA-UE) was found to be strongly correlated with AUL magnitude (r = 0.82) [34] (r = 0.7) [42] and moderately to strongly correlated with arm ratio measures, both magnitude (r = 0.82) [34] (r = 0.59) [42] and use time (r = 0.85) [24]. A moderate correlation was also found between its lower extremity scale (FMA-LE) and the leg magnitude ratio (r = 0.61) [34]. These results indicate that higher sensor-based symmetry values are related to better performance. Strong correlations were found between magnitude ratio and the Upper Extremity- and Hand-subscales of Brunnstrom Recovery Stage (BRS), and the Simple Test for Evaluating Hand Function (STEF)—affected side (r = 0.77, r = 0.71, r = 0.86, respectively) [10]. Furthermore, moderate to strong correlations were found between unilateral magnitude and the STEF (r = 67 AUL, r = 0.75 UUL) [10]. The rest of the correlations between clinical scales and sensor-based metrics were either low or moderate [10,22,27,35,38,42]. 

## 4. Discussion

The aim of this review was to provide an overview of the literature describing setups used to measure the quantitative and qualitative aspects of movements of stroke patients under free living conditions using wearable sensors. Additionally, we evaluated the relation between the sensor-based outcomes that were obtained from moving in a free-living environment and the level of functioning as assessed by existing clinical evaluation methods. Based on the results of our review it appears that continuous monitoring of motor function of stroke patients during their activities of daily living using wearable sensors is feasible, and an overview of setups is provided. The sensor-based outcomes showed weak to strong correlations with the scores on clinical scales assessing motor functioning.

In comparison to earlier reviews related to our topic, we considerably updated the discussed literature and added a structured overview of setups used to measure the quantitative and qualitative aspects of movements of stroke patients under free living conditions using wearable sensors. Noorkõiv et al. [12] included eight studies in their review, of which five [5,24,27,28,41], are also included in ours. Johansson et al. [13] included 24 studies in stroke, of which eight [20,23,24,25,26,27,28,40] are also included in ours. Studies that were discussed in earlier reviews, but not in ours, mostly either didn’t focus on free-living conditions, or discussed physical activity instead of quantitative or qualitative aspects of movement. Some of these earlier reviews [12,14], are also explicitly limited to studies that used accelerometry or accelerometry-based proprietary devices such as actigraphs, actiwatches or step counters, probably because the use of gyroscopes or combinations of sensors in IMUs is more recent.

The studies included in the current review made use of different wearable sensors to measure movements of stroke patients in free living conditions. All included studies used at least 3D accelerometer sensors. Reliability of accelerometer derived movement measures has been addressed extensively in numerous laboratory studies. Until recently, the technology necessary for long term measurements was not available. However, due to technological advancements, accelerometers are now easily available at low cost and with low power consumption, making long term data collection feasible. This might explain why all studies included in the present review, derived measures from at least accelerometers, to quantify movements during activities of daily living. Inertial measurement units (IMUs), combining accelerometers with other sensors, can provide more specific information about the patient’s movements, because by integrating information from 3D accelerometers, gyroscopes and magnetometers, body segment orientation and joint angles can be calculated [50]. Considering technological developments concerning miniaturization and power consumption, we expect to see a further increase in the number of studies of movement in daily life using IMUs over the next years. Custom made equipment based on newer and improved sensor technology may also provide an alternative to the monitoring of factors that were impossible to quantify until now, such as the ‘manumeter’ [36] which allows the quantification of hand movements during ADL. Another type of custom system used in one of the studies included in this review, is the electrohydraulic activity sensor [29,44]. This sensor was used to provide information about the elevation of the forearm relative to the body. In principle, for this specific purpose IMUs can be used also [31], which has advantages in terms of wearability, usability and compliance. However, when the aim is to accurately measure isolated upper limb multi joint movements, and the coordination between the joints, single-sensor systems might not be adequate. More complex sensor configurations, with additional sensors attached to each segment of the arm, as well as to the trunk, will definitely improve the analysis of such multi-joint movements [19]. 

The location of the sensors was quite similar in the studies that focused on the measurement of upper body movement. All studies used accelerometers on both wrists, except for the studies using the most extensive measurement set-ups. The studies with electrohydraulic sensors [29,44] needed setups covering the whole arms and some studies using IMUs [31,32] needed additional sensors on the whole body to create a full-body kinematic model. While this may provide more information compared to the use of only accelerometers on the wrist (and sometimes sternum, waist and/or lower limb), the more complex setup needed when using multiple IMUs may reduce usability and long-term compliance. While the more technologically advanced IMUs may eventually replace single 3D accelerometers, the use of single 3D accelerometer sensors, that require less power consumption and processing time, on one or only a few body positions allowing for easy use, appears to be a good alternative for long term monitoring of upper limbs during daily living. 

For the identified studies of gait during ADL, only accelerometers were used, located on the sternum, lower back, upper leg and/or lower leg, Accelerometers at these locations have excellent to good validity and reliability for spatiotemporal gait measures, but for measures of quality of gait this depends on the measurement protocol, algorithms and design [51]. With respect to sensor locations, with the appropriate correction methods, vertical and anterio-posterior accelerations were found to be reliable for back and shank sensor locations in osteoarthritis patients [52]. For stroke patients in a controlled setting, a sensor on the back was found to give reliable outcomes with respect to gait asymmetry [52,53,54]. However, reliability has not been assessed for stroke patients during long-term measurements in unsupervised settings. 

Our overview of the reported correlations between sensor-based measures of movement and clinical scale scores suggests that the sensor-based measures represent a different construct compared to clinical scales thus providing additional information. After all, a perfect correlation between sensor-based measures and clinical scale scores would indicate that sensors and clinical scales provide the exact same information. In the studies included in this review, however, the correlation is mostly weak to moderate. This thus implies that with sensors, additional information is gained on top of the information gained from clinical scales. With patient reported outcome measures, insight into how patients experience their functioning and limitations is gained, while sensors provide objective information about (ADL) functioning. Previous research has shown that these are indeed two different constructs with patient reported outcomes being influenced by e.g., amount of pain experienced during ADL [55]. Both, however, provide information that is valuable and important to improve the care of this patient group.

### Limitations and Future Recommendations

Quality of included studies was mostly good, with only three studies being of moderate quality (9%). Lowest scores were found for items related to corrections for or assessment of confounding variables, and participant inclusion bias. The latter is a common issue in patient studies; usually participants are not recruited from the general population. Furthermore, only studies written in English were included. While we selected studies for this review based on well-defined selection criteria, it was very difficult to combine results from multiple studies. We found a disparity among studies in time measured after stroke, the units in which the outcomes were reported (e.g., actigraph counts vs. homemade counts vs. g/min), the length of the measurement, the lack of transparency of algorithms to calculate parameters, differences in design and methodology as well the use of a wide variety of clinical scales to assess upper extremity functioning in stroke patients (see Figure 3). This underlines the need for clear protocols, guidelines and transparency of algorithms, with respect to the use of wearable measurement systems and the extraction of metrics from these systems when monitoring stroke patients outside the clinic. Also, studies should avoid reporting their findings in proprietary, closed-source units. When reporting time-based outcomes, fraction or percentage of time should be prioritized over absolute values in hours. We also encourage future studies to report the numerical value of the measures extracted during their experiments, even if it’s not one of the aims of their study, as it could help with future pooling and meta-analysis of such measures.

Thanks to technological developments, in the near future, more sensors will become available that are “wearable”, precise and fast enough for monitoring in free living conditions. For example, continuous monitoring using insole pressure sensors, instead of the use of force plates which until now have been used in controlled settings [55], could provide information about the symmetry and loading patterns of hemiparetic gait during activities of daily living (ADL). Wearable wireless electromyography could help to understand balance impairments, loading and compensatory activity during ADL, for walking as well as upper extremity function, providing directions for interventions [56]. Similarly, muscle activity patterns of walking might provide important information about compensatory abilities and intervention strategies [57,58]. Modern barometric pressure sensors, which are fast, low in noise and can detect height differences as little as 10 cm should also be considered. While some studies already used them to help improve activity classification for stroke patients [59,60], to our knowledge no current study has used these to extract measures of movement in stroke patients. This kind of sensor has already demonstrated very good results, combined with accelerometers, in estimating Timed-up-and-go (TUG) scores from ADL data in hip arthroplasty patients [61].

## 5. Conclusions

Continuous monitoring of motor function of stroke patients during their activities of daily living using wearable sensors is in principle feasible and provides complementary information to clinical assessments. With recent advances in technology the options, regarding the type of sensors that can be used, increase, as well as the accuracy and possibilities of extracting different metrics from them. Sensor-based measures of movement provide additional information in relation to the scores on clinical scales assessing motor functioning and both are needed to gain better insight in patient behavior and recovery. However, there is a strong need for standardization and consensus, regarding clinical assessments, but also regarding the use of specific algorithms and metrics for unsupervised measurements during daily life. Lab protocols and metrics, cannot simply be generalized to unsupervised settings.

## Supplementary Materials

The following supporting information can be downloaded at: https://www.mdpi.com/article/10.3390/s22031050/s1, Table S1: Downs_&_Black_Checklist.

## Appendix A

**Table A1:** Continuous recording in the hospital or during rehabilitation.

Reference	Experimental Design	Sensor & Placement	Measurement Task	Population (Mean ± std)	Clinical Measures	Sensor Based Measures	Results
Held et al. [31]	Observational, prospective cohort study	14 IMU’s: triaxial ACC, magneto-meter and gyroscope. (Xsens suit) Feet, shoulders lower + upper legs, lower + upper arms, sternum, sacrum	Recording during clinical assessments + 3 h recording during ADL, at: T1: 2 wks. before discharge T2: right after discharge T3: 4 wks. after discharge	Stroke: N = 4 Age: 48–55 y TAS: 5.25 ± 4.08 m. Mild-to-severe UL impairment (FMA-UE, ARAT)	FMA-UE, ARAT	Average joint RoM in elbow flexion, shoulder abduction, and shoulder flexion (AUL) Reaching area of AUL Reaching counts of AUL Ratio of UUL-AUL reaching counts	Clinical assessment score showed no sign. improvement after discharge. Wide variability among participants. In general, AUL motor function improved during inpatient rehabilitation, but declined between T2 and T3 Reaching counts: increased on avg. from 63 (T1) to 202 (T3) Ratio of reaching counts: increased on avg. 26.8% (from T1 to T3)
Held et al. [32]	Observational, prospective cohort study	See [23]	See [23]	See [23]	See [23]	Reaching counts of AUL Maximum reaching distance of AUL	Reaching counts (exact value not available): increased in all participants from T1 to T2. Maximum reaching distance (exact value not available) higher during clinical assessment compared to daily life **.
Iacovelli et al. [33]	Observational, Cross-sectional	Triaxial ACC (EZ430-Chronos, Texas Instruments) Both wrists	24 h continuous recording in clinic	Stroke: N = 20 Age: 69.2 ± 10.1 y TAS: 3.3 ± 1.6 d, AcuteHealthy control: N = 17 Age: 70.4 ± 7.3 y	(SMS-)NIHSS	MAe1_24 h = SD of the acc norm. MAe2_24 h = norm of the SD of the acceleration axis. AR1_24 h Asymmetry Rate Index of MAe1_24 h AR2_24 h Asymmetry Rate Index of MAe2_24 h	MAe1_24 h and MAe2_24 h: AUL < UUL *** AR1_24 h: Stroke −70.5% ± 68.7% Controls −39.2% ± 44.6% AR2_24 h: Stroke −83.2% ± 92.1% Controls −21.1% ± 20.2% Discarding passive movements, AR2_24 h with NIHSST1: r = 0.714 ***, CI 95% = 0.42–0.90, AR2_24 h with SMS-NIHSST1: r = 0.812 ***, CI 95% = 0.62–0.96. With passive movements AR2_24 h with NIHSST1: r = 0.408, ns, CI 95% = 0.03–0.73) AR2_24 h with SMS-NIHSST1: r = 0.546 *, CI 95% = 0.23–0.79
Lucas et al. [43]	Observational, Cross-sectional	Triaxial ACC (Axtivity AX3) Both wrists, both ankles	>7 d continuous recording in hospital	Stroke: N = 4 Age: 51.7 ± 13.2 y TAS: Not reported (Acute)	Oxford Grading Motor Scale	Acc norm (avg, min, max). Movement smoothness (signal jerk). Power and Frequency of the 1st and 2nd dominant FFT coefficient. Number of events per hour.	The SVM classified “dependent” and ‘‘antigravity’’ limbs (0–2 and 3–5 oxford grading motor score, respectively) with performance significantly above baseline in most instances *
Narai et al. [10]	Observational, Cross-sectional	Triaxial ACC (Air Sense) + Uniaxial ACC (Lifecorder EX4) Triaxial ACC: both wrists Uniaxial ACC: waist	24 h continuous recording in clinic	Stroke: N = 19 Age: 77 ± 6 y TAS: 17 ± 7 d	MAL-AOU and -QOL NIHSS BRS STEF FIM	Movement counts (1 min integration of band-passed acc norm minus gravity) of AUL, UUL Movement count ratio of AUL-UUL Delta counts of AUL–UUL	AC per h during 24 h: AUL 1332 ± 644 UUL 1734 ± 914 * During 12 h (daytime): AUL 2123 ± 792, UUL 2730 ± 1069 * Correlation Movement counts, Ratio of AUL-UUL, Delta counts AUL-UUL: MAL-AOU r = 0.58, 0.84, 0.70 MAL-QOM r = 0.55, 0.79, 0.66 NIHSS r = −0.69, −0.60, −0.27 BRS-UE r = 0.64, 0.77, 0.52 BRS-H r = 0.57, 0.71, 0.56 STEF -AS r = 0.67, 0.86, 0.74 STEF-US r = 0.75, 0.54, 0.28 FIM-M r = 0.70, 0.50, 0.17 FIM-C r = 0.61, 0.39, 0.13 resp.
Prajapati et al. [21]	Observational, Cross-sectional	Triaxial ACC (Sparkfun Electronics) Both Ankles	8 h continuous recording in hospital, including therapy	Stroke: N = 16 Age: 59.7 ± 15.3 y TAS: 37.8 ± 24.7 d	CMSABBS	Walking time. Duration of walking bouts. Symmetry (swing time ratios).	Walking time: 47.5 min ± 26.6 min Number of walking bouts: 57.7 ± 30.5, duration: 54.4 s ± 21.5 s Decreased gait symmetry during free-living recordings compared to clinic **
Rand & Eng [5]	Observational, prospective cohort study	Triaxial ACC(Actical) Both wrists, on the hip	Stroke: 3 d continuous recording at: T1: first rehabilitation week, T2: 3 weeks after start rehabilitation. Healthy controls: 5 d continuous recording.	Stroke ambulant: N = 27Age: 64.3 ± 13.4 y TAS: 33.3 ± 19.2 dStroke Wheelchair users: N = 33Age: 58.2 ± 12.8 yTAS: 33.5 ± 22.1 d Healthy Controls: N = 40 Age: 71.3 ± 3.8 y	FMA-UE, ARAT BBT 10 MWT 6 MWT FIM	Mean daily use: Lower body (total steps/3), for entire day, during therapy, entire day minus therapy. Upper body ((AC except walking time)/3)	Number of steps per day (median, IQR): Stroke ambulant T1 306 (82–3095) T2 1423 (178–3183) Stroke wheelchair users T1 143 (71–386) T2 283 (80–1650) All stroke T1 176 (78–1891) T2 302 (96–2315). Controls 5202 (3548–6333) Walking during therapeutic sessions accounted for 12% (T1) and 26% (T2) of entire day AC in ST. Upper limb AC (median, IQR): Stroke: T1 AUL 35,734 (18,167–84,238) UUL 147,500 (90,477–224,835) T2 AUL 41,541 (19,340–105,980) UUL 164,875 (95,287–212,920) Controls: DUL 184,761 (131,523–241,819) NDUL 159,698 (107,826–217,489)
Urbin et al. [25]	Observational, prospective cohort study	Triaxial ACC (GT3Xþ, ActiGraph) Both wrists	22 h continuous recording: T1: after pre and T2: posttest inpatients T3: after 24th training session + recording during 24th training session (outpatients).	Stroke inpatients: N = 8 Age: 56 ± 10.4 y TAS: <30 d Stroke outpatients: N = 27 Age: 62 ± 9.4 y TAS: >6 m	ARAT NIHSS	AUL-UUL: - Time use ratio - magnitude ratio - variation ratio AUL: - median acc norm. - variability Bilateral median Bilateral variability	Five metrics improved from T1 to T2, all *. AUL-UUL use ratio: 0.54 ± 0.18 to 0.86 ± 0.28 magnitude ratio 0.24 ± 0.32 to 0.71 ± 0.65 variation ration 0.60 ± 0.23 to 0.80 ± 0.22 AUL median 0.05 ± 0.09 to 0.23 ± 0.21 variability 0.53 ± 0.16 to 0.72 ± 0.19 T3 AUL-UUL ratios and AUL outcomes significantly higher, all ***, during the training session compared to free-living environment (exact values not available). Correlation between ARAT and Time use ratio: rho = 0.79 *** magnitude ratio rho = 0.83 *** variation ratio rho = 0.85 *** AUL median rho = 0.75 *** Variability rho = 0.73 *** Bilateral median, variability n.s.
Sanchez et al. [23]	Observational, prospective cohort study	Uniaxial ACC (ADXL202, Analog Devices) 1 on both thighs, 3 on sternum	8 h continuous recording at: T1: TAS 1 w T2: TAS 12 w T3: TAS 48 w	Stroke: N = 23 Age: 58.13 ± 12.58 y TAS: ~1 w Healthy Controls: N = 20 Age: 55.35 ± 12.70 y	--	Amount outcomes: percentage of time walking, standing, sitting, lying and sedentary. Distribution outcomes: number and mean duration of walking bouts, walking coefficient of variation, sedentary exponent. Quality outcomes: step regularity, gait symmetry, stride regularity, step-time ratio (affected/unaffected), walking speed	% of time Walking T1: 3.35% ± 2.19 T2: 12.45% ± 2.37 ** Standing T1: 8.25% ± 3.65 T2: 27.14% ± 3.15 ** Sedentary T1: 87.37% ± 4.27 T2: 70.07% ± 3.19 ** T2 to T3 n.s. change Number of walking bouts T1: 26.29 ± 12.85, T2: 103.87 ± 12.28 ** T2 to T3 n.s. change Sternum sensor improvement of Gait symmetry T1: 1.12 ± 0.04 T2: 1.03 ± 0.04 T3: 1.02 ± 0.04 (T1–T3 *) Step regularity T1: 0.48 ± 0.06 T2: 0.59 ± 0.05 T3: 0.64 ± 0.05 (T1 -T3 *)
Thrane et al. [24]	Observational, Cross-sectional	Uniaxial ACC (ActiGraph GT1M) Both wrists	24 h continuous recording. Car driving, sleeping data excluded.	Stroke: N = 31 Age: 65 ± 14 y TAS: 10.6 ± 6 d	FM Sunnaas ADL-index 5STS NIHSS	UL use time Arm movement ratio	Average UUL use: 4.5 h ± 1.7 Average AUL use: 3 h ± 1.7 h Arm movement ratio: 1.5 (1.1–2.0) (Median, IQR) Correlations between 5STS and FMA rho = −0.529 ** AUL use time rho = −0.627 *** Arm movement ratio rho = −0.643 *** Correlations between FMA and AUL use time rho = 0.601 *** Arm movement ratio rho = −0.851 *** Latter supported by regression analysis: β = −0.05 ***.
Waddell et al. [30]	Observational, prospective cohort study	Triaxial ACC (Actigraph Link) Both wrists	24 h continuous recording at T1: TAS 2 w T2: TAS 4 w T3: TAS 6 w T4: TAS 8 w T5: TAS 12 w	Stroke: N = 22 Age: 68.7 ± 9.9 y TAS: <2 w	ARAT MoCA SAFE	Hours of AUL use AUL-UUL use ratio Magnitude ratio (zero-centered, negative values imply greater UUL movement) Bilateral magnitude	High variability in UL performance across participants for all metrics. But it was shown that performance increased significantly (*p* value not reported) on the first 12 weeks after stroke. T1: Hours of AUL use 2.82 ± 1.8 h AUL-UUL use ratio 0.52 ± 0.26 Magnitude ratio −4.5 ± 2.9 Bilateral magnitude 72.5 ± 16.9 Estimated slope values (estimates rate of change per 2 weeks for the study duration) of the hierarchical linear model for the entire sample: Hours of AUL use: 0.17 ± 0.04 *** Use ratio 0.02 ± 0.004 *** Magnitude ratio 0.23 ± 0.05 *** Bilateral magnitude 1.46 ± 0.49 **
Andersson et al. [34]	Observational, Cross-sectional	5 triaxial ACC. (Shimmer 3) Trunk, both wrist, both ankles	2 sessions of 48 h recording in a rehabilitation clinic. Only daytime activity (8 h-20 h) was used.	Stroke: N = 26 Age: 55.4 ± 11.9 y TAS: 56 ± 24 d. Mild-to-severe impairment (FMA-UE/LE)	FMA-UE, FMA-LE, modified Ashworth Scale, 10 MWT, MRS	Arm and leg activity (Signal magnitude area) Arm and leg activity ratio	Sensor based measures correlated with with clinical measures: AUL activity, FMA-UE r = 0.82 **** AUL activity, 10 mWT r = 0.79 **** Affected leg activity, 10 mWT r = 0.77 **** Arm activity ratio, FMA-UE r = 0.82 **** Leg activity ratio, FMA-LE r = 0.61 **** Leg activity ratio, 10 mWT r = 0.52 ****
Reale et al. [38]	Observational, prospective cohort study	Two triaxial acc. (EZ430-Chronos, Texas Instruments) Both wrists	T1 (TAS 48–72 h): 24 h continuous recording T2 (TAS 90 d): MRS evaluation	Stroke: N = 20 Age: 69.2 ± 10.1 y TAS: 48–72 h	NIHSS MRS ASPECTS	Asymmetry Rate Index for the 24 h period (AR2_24 h)	AR2_24 h parameter during the acute phase value of MRS at 90 d r = 0.69 **** AR2_24 h > 32% during acute phase predicts a poorer outcome (RS > 2 at 90 d), with sensitivity = 100% and specificity = 89%
Regterschot et al. [39]	Observational, prospective cohort study	3 triaxial acc. (Activ8 Activity Monitor) Both wrists and the thigh of the nonaffected leg	Continuous recording for one week (only during walking hours for the wrist sensors) At: T1: TAS 3 w (rehabilitation center) T2: TAS 12 w (rehabilitation center or home depending on the patient) T3: TAS 26 w (home)	Stroke: N = 33 Age: 57.3 ± 8.5 y TAS: 3 w (NIHSS 5 A/B or 6 A/B 4 ≥ score > 0)	NIHSS FMA-UE	Using the thigh sensor to select only sitting and standing periods, mean daily values for: Total AUL AC Total UUL AC Ratio of AUL / UUL AC Mean AUL AC per sit/stand hour. Mean UUL AC per sit/stand hour.	Change in time of the sensor measures: Total AUL AC per day: T1 to T2 = +30% * Total UUL AC per day: T1 to T2 = −13% **** T1 to T3 = −22% **** Total UUL AC per day: T1 and T2 = −13% **** T1 and T3 = −22% **** AUL / UUL AC ratio T1 to T2 = +43% **** T1 to T3 = +95% **** Mean AUL AC per sit/stand hour T1 to T2 = +31% **** T1 to T3 = +48% * Mean UUL AC per sit/stand hour. T1 to T3 = −18% ***
Le Heron et al. [40]	Observational, prospective cohort study	Triaxial ACC (Crossbow Imote2) Both wrists	1 h minimal recording in clinic at: T1: 54 h (median, 47–100) T2: T1 + 24 h.	ST: N = 20 Age: Median 77 y, IQR 59–82 y TAS: (T1) 54 h (median, range 47–100) Mild-to-moderate stroke severity (NIHSS) HC: N = 10 Age: Median 64 y, IQR 48–71 y	(SMS-)NIHSS	ICC of time-matched series of Acc. spectral power for both arms.	Correlation between NIHSS at T1 and the magnitude of ICC: rho = −0.53 *. The optimal diagnostic threshold for ICC magnitude was 0.7. At this threshold, ROC curve analysis using the ICC magnitude to distinguish stroke patients from controls yielded an AUC of 0.84
Gebruers et al. [42]	Observational, prospective cohort study	Triaxial ACC (ambulatory monitoring). Both wrists.	T1: (<1 w after stroke) At least 24 h continuous recording. T2: T1 + 3 m, MRS assessment.	Stroke: N = 129 Age: 70 ± 11.4 y TAS: <1 w, median 1 d	NIHSS, FMA-UE (T1, T2), MRS (T2)	AUL AC AUL/UUL AC ratio	Correlation between: FMA-UE (T1): FMA-UE (T2) r = 0.69 * MRS: r = −0.66 * AUL AC: FMA-UE (T2) r = 0.70 * MRS: r = −0.60 * AUL/UUL AC ratio: FMA-UE (T2) r = 0.59 * MRS: r = −0.48 *

Statistical significance levels: n.s. not significant; * *p* < 0.05; ** *p* < 0.01; *** *p* < 0.005; **** *p* < 0.001. Abbreviations in alphabetical order: AC = activity counts, ACC = accelerometer, ADL = Activities of Daily Living, AUL = affected upper limb, d = day(s), DUL = Dominant Upper Limb, h = hour(s), IMU = Inertial Measurement Unit, NDUL = Non Dominant Upper Limb, SVM = Support Vector Machine, ST = Stroke (patient), TAS = Time After Stroke, UL = Upper Limb, UUL = Unaffected Upper Limb, y = year(s), w = week(s). Clinical assessments in alphabetical order:: 5STS = 5 Times Sit to Stand Test, 6 MWT = 6 min walk test, 10 MWT = ten meters walk test, ARAT = Action Research Arm Test, ASPECTS = Alberta Stroke Program Early CT Score, BBT = Box and Blocks Test, BBS = Berg Balance Scale, BRS(-UE, -H) = Brunnstrom Recovery Stage (Upper Extremity, Hand), CMSA = Chedoke McMaster Stroke Assessment Scale, FIM (-M, -C) = Functional Independence Measure (Motor, Cognitive), FMA(-UE) = Fugl-Meyer Assessment (Upper Extremity), MAL(-AOU, -QOM) = Motor Activity Log (Amount of Activity, Quality of Movement), MoCA = Montreal Cognitive Assessment, (SMS-), MRS = Modified Rankin Scale, NIHSS = (Supplementary Motor Scale) National Institute of Health Stroke scale, SAFE = shoulder abduction-finger extension, STEF (-AS, -US) = Simple Test for Evaluating Hand Function (Affected Side, Unaffected Side), Sunnaas = Sunnaas ADL-index for personal (self-care) ADL activities.

**Table A2:** Continuous recording during Activities of Daily Living (ADL).

Reference	Experimental Design	Sensor & Placement	Measurement Task	Population (Mean ± std)	Clinical Measures	Sensor Based Measures	Results
Bailey et al. [8]	Observational, Cross-sectional	Triaxial ACC (GT3X+ Actigraph) Both wrists	24 h continuous recording during ADLHealthy vs. Stroke	Stroke: N = 48 Age: 59.7 ± 10.9 y TAS: >6 m ARAT = 31.3 ± 11.9 Healthy Controls: N = 74 Age: 54.3 ± 11.3 y	--	Bilateral acc norm Magnitude ratio of AUL-UUL Total duration of unilateral activity Total duration of (simultaneous) bilateral UL activity Total duration of UL activity	Bilateral acc norm (1 s epoch) Median IQR Stroke: 82.4 (27.6), Controls: 136.2 (36.6) *** Magnitude ratio Stroke: −2.2 (6.2) *** Controls: −0.1 (0.3) *** (Median, IQR) Duration of unilateral activity S Stroke: UUL = 3.4 ± 1.2 h vs. Controls: DUL = 1.9 ± 0.5 h *** Stroke AUL = 0.8 ± 0.5 h vs. Controls NDUL = 1.5 ± 0.5 h *** Bilateral activity Stroke 4.1 ± 1.7 h Controls: 7.2 ± 1.9 h *** Total UL activity Stroke 8.4 ± 2.2 h Controls 10.7 ± 2.1 h ***
Chen et al. [9]	Observational, prospective cohort study	ACC (MicroMini-Motionlogger, Ambulatory Monitoring) Both wrists	72 h continuous recording during ADL, except when bathing (T1) Pre and (T2) post 4-week rehabilitation intervention	Stroke: N = 82 N = Age: 55.3 ± 10.71 y TAS: 20.46 ± 13.43 m Mild-to-moderate UL impairment	MAL-AOU MAL-QOM SIS (physical function subscale) NEADL	AUL AC (Action4 software)	Mean AUL activity counts (1 m epoch): T1 3701.8 ± 1447.2 T2: 4247.2 ± 1549.6 Predictive validity of AUL activity compared to MAL-AOU r = 0.47, Physical function of SIS r = 0.42 NEADL r = 0.34 MAL-QOM r = 0.57; all ** Responsiveness of AUL T1 vs. T2: SRM = 0.72 Minimal clinically important difference using anchor-based methods and MAL-AUM 751.58 (28%) MAL-QOM, 631.06 (28%) SIS, 574.94 (29%) NEADL 614.36 (13%)
Leuenberger et al. [19]	Observational, Cross-sectional	5 IMU: triaxial ACC, gyroscope and magneto-meter (unused) + Barometric pressure sensor (unused) (ReSense) Both wrists, both shanks, around waist. Recordings from waist IMU were excluded	48 h continuous recording during ADL	Stroke: N = 10 Age: 52.7 ± 13.6 year TAS: 21.6 ± 10.6 w	BBT	Mean UL ACs (formula provided) p/min during awake time, including or excluding walking Ratio AUL-UUL AC Duration of AUL use Normalized probability distribution of forearm elevation Total gross arm movements duration during the recording period.	Correlation between ratio of AUL AC and BTT incl. walking r = 0.69 * excl. walking r = 0.93 *** Correlation between ratio of AUL-UUL AC and ratio of AUL-UUL BTT incl. walking r = 0.49 *; excl. walking r = 0.84 *** Correlation between duration of AUL use and BTT: incl. walking r = 0.77 ** Correlation between AUL BBT and difference between forearm elevation probability distribution’s means UUL and AUL Excl. walking r = 0.68 * Correlation between BTT and gross arm movement duration: Inc. walking r = 0.95 *** Exc. walking r = 0.97 *** Correlation between ratio of AUL-UUL BTT and ratio AUL-UUL of gross arm movement duration: Inc. walking r = 0.95 *** Exc. walking r = 0.9***
Michielsen et al. [20]	Observational, Cross-sectional	Uniaxial ACC + Biaxial ACC Both wrists. Waist	24 h continuous recording during ADL	Stroke: N = 38 Age: 56.6 ± 12.6 y TAS: 4.5 ± 3.2 y Healthy Control: N = 18 Age: 48.1 ± 10.9 y	--	Duration of activities as % of 24 h period. UUL, AUL–Stroke subjects, DUL, NDUL–healthy subjects expressed as: - Time - Time while sitting or standing - Mean intensity (g/min) of the period of uni- or bimanual use	Daily activities stroke vs. healthy subjects: Dynamic (7% vs. 10%), sitting (39% vs. 38%), standing (9% vs. 15%), lying (45% vs. 37%). Stroke vs. Healthy: UUL vs. AUL use 5.3 h vs. 2.4 h ** DUL vs. NDUL use 5.1 h vs. 5.4 h * During sitting: AUL vs. NDUL use1.3 h vs.2.6 h ** During standing: Stroke used both limbs less than healthy: AUL vs. NDUL 1.1 h vs. 2.4 h ** UUL vs. DUL 1.8 h vs. 2.5 h * Both groups UL use is highest during bimanual activities. Unimanual activities Stroke vs. Healthy intensity AUL vs. NDUL: 0.02 g/min vs. 0.04 g/min intensity UUL vs. DUL: 0.04 g/min vs. 0.04 g/min
Punt et al. [11]	Observational, Cross-sectional	Triaxial ACC (McRoberts) Both ankles	7 d continuous recording during ADL	Stroke: N = 40 of which Fall: N = 15 Age: 64.6 ± 8.5 y TAS: 113 ± 109 m NFall: N = 25 Age: 58.4 ± 14.3 y TAS: 71.8 ± 65 m	10 MWT TUG BBS	Gait characteristics: Gait speed (m/s) Stride time (s) Standard deviation of accelerations. Harmonic ratio Index of harmonicity Amplitude of the power dominant peak Width of the power dominant peak Local divergence exponent	ADL gait characteristics predicted falls, AUC = 0.72, better than clinical assessments, AUC = 0.64. Sign *. differences between NFall and Fall in Gait speed NFall 0.73 ± 0.16 Fall 0.62 ± 0.12 SD of acc. in V direction: NFall 1.63 ± 0.52 Fall 1.23 ± 0.39 SD of acc. in AP direction: NFall: 1.38 ± 0.33 Fall: 1.16 ± 0.23 Harmonic ratio in AP direction: NFall: 1.13 ± 0.19 Fall: 1.00 ± 0.19 Index of harmonicity in M direction: NFall: 0.42 ± 0.20 Fall: 0.57 ± 0.26 Amplitude of the power dominant peak in medio-lateral direction: NFall: 0.44 ± 0.16 Fall: 0.57 ± 0.24 Others n.s.
Rand & Eng [22]	Observational, prospective cohort study	Triaxial ACC (Actical) Both wrists	3 d continuous recording at: T1: 4 w T2: 12 m	Stroke: N = 32 Age: 58.1 ± 12.4 y TAS (T1): 29.6 ± 15.5 d	MAL-AOU FMA-UE ARAT TL BBT Grip strength	Mean AC AUL	Scores on all clinical assessments except MAL, improved significantly from T1 to T2 ***. Mean AC (median, IQR) at T1: AUL 43,030 (21,360–112,865) UUL 171,610 (106,660–228,592) T2: AUL 53,916 (22,353–106,693) UUL 152,065 (58,921–212,435) Correlation between MAL and AUL AC at T1: r = 0.65 ** T2: r = 0.57 ** AC and MAL at T2 correlated with different factors, but are both independent of AUL dominance, cognition and depressive symptoms: AC: Age r = 0.50 *** FMA-UE r = 0.56 *** ARAT r = 0.59 *** Grip r = 0.50 ** BBT r = 0.62 *** TL r = −0.51 ** MAL: Gender r = 0.369 * FMA r = 0.66 *** ARAT r = 0.78 *** Grip r = 0.7 *** BBT r = 0.81 *** TL r = −0.51 **
Uswatte et al. [26]	Observational, prospective cohort study	Biaxial ACC (71256, ActiGraph) Both wrists, less impaired side of the chest, more impaired ankle.	72 h continuous recording at: CIMT rehabilitation group: (T1) Pre and (T2) post CIMT rehab (2 w).Normal rehabilitation group: T2 = T1 + 2 w.	Stroke CIMT: N = 10 Age: 61.4 ± 20.0 y TAS: >1 y Stroke normal rehabilitation group: N = 10 Age: 63.7 ± 13.5 y TAS: >1 y UL impairment: Mild to moderate (N = 19), moderate to severe (N = 1)	MAL-QOM	Mean ratio AUL-UUL use	Intervention group: increase from T1 to T2 (mean change 0.08 ± 0.09*). Control group: non-significant change. Correlation between ratio and: MAL-QOM score at T1: r = 0.74 *** T1 to T2: r = 0.71 ***
Uswatte et al. [27]	Observational, prospective cohort study	Biaxial ACC Both wrists	See 24	Stroke intervention: N = 82 Age: 63.0 ± 12.8 y TAS: >1 y Stroke control: N = 87 Age: 64.2 ± 12.7 y TAS: >1 y Mild to moderate UL impairment	AAUT MAL-QOM SIS	Mean duration of AUL use (%) (2) Mean ratio AUL-UUL use	T1: Mean duration % AUL use Intervention group 21.7 ± 9.4 Control group 22.5 ± 11.2 All: 22.1 ± 10.3 Mean Ratio AUL-UUL Intervention group 0.56 ± 0.15 Control group 0.57± 0.17 All 0.56 ± 0.16 N.s. from T1 to T2 (*p* > 0.48) Correlation between ratio AUL-UUL use and MAL-QOM r = 0.52 * AAUT r = 0.60 * SIS r = 0.16 ***
van der Pas et al. [28]	Observational, Cross-sectional	Triaxial ACC (Actiwatch AW7) Both wrists	60 h continuous recording during ADL	Stroke: N = 45 Age: 59.4 ± 9.2 y TAS: 2.0 ± 1.6 y	MAL-AOU MAL-QOM SIS-mobility SIS-hand function	Unilateral arm activity relative to waking hours. Bilateral arm activity relative to waking hours. Ratio of UUL-AUL use	Activity Counts AUL AC 7501 ± 3370 UUL AC 16756 ± 4836 Bilateral AC 12129 ± 3782 Ratio of UUL-AUL 0.69 ± 0.10 Correlation between unilateral arm activity and MAL-AOU r = 0.58 *** MAL-QOM r = 0.65*** Correlation between bilateral arm activity and MAL-AOU r = 0.37 ** MAL-QOM r = 0.43 *** Correlation between ratio of UUL-AUL and MAL-AOU r = 0.60 *** MAL-QOM r = 0.66 *** Correlation between unilateral arm activity and SIS-hand function r = 0.61 *** SIS-mobility r = 0.41 ** Correlation between bilateral arm activity and SIS-hand function r = 0.43 *** SIS-mobility r = 0.39 ** Correlation between ratio of UUL-AUL and SIS-hand function r = 0.58 *** SIS-mobility r = 0.23 n.s.
Vega-Gonzalez et al. [44]	Observational, Cross-sectional	Electrohydraulic activity sensor (SULAM system) Both upper limbs	8 h continuous recording during ADL	Stroke: N = 10 Age: 55–79 y TAS: >1 y Healthy Controls: N = 10 Age: 23–57 y	--	Use ratio. Bimanual movement time. Unimanual movement time. Movement time (unimanual, bimanual) per region: below waist (below-W), between waist and chest (W-to-C), between chest and shoulder (C-to-S), between shoulder and head (S-to-H), above head (above-H).	User Ratio ST’s UUL use was “twice as much” as AUL use *** (exact amount not reported). Controls’s DUL use 10% higher than NDUL use ***. Bimanual movement time > for Controls than Stroke *** (exact values not reported). N.s. difference for unimanual movement time between HC and ST Stroke UUL use was > AUL use in the ranges W-to-C: 20 h vs. 11.7 h **** C-to-S: 7.8 h vs. 2.2 h * HC’s DUL use was > NDUL use in the ranges C-to-S: 13 h vs. 9.2 h **** S-to-H 1.7 h vs. 0.9 h * n.s. difference for the other ranges
Vega-González & Granat [29]	Observational, Cross-sectional	Electrohydraulic activity sensor (SULAM system) Both upper limbs	8 h continuous recording during ADL	Stroke: N = 10 Age: 56–80 y TAS: >1 y Healthy Controls: N = 10 Age: 22–35 y	--	Use ratio Bimanual movement time. Unimanual movement time. Composite movement time (bimanual + unimanual). Movement time per region: below midtrunk (below-MT), between midtrunk and shoulder (upper-T), above shoulder (above-S). Distance above shoulder.	User Ratio Stroke UUL vs. AUL use: 2.55 h ± 0.74 vs. 0.91 h ± 0.54 *** Control DUL vs. NDUL use: 2.98 h ± 0.55 vs. 3.69 h ± 0.37 *** Bimanual movement time Stroke 0.80 h ± 0.49 vs. Controls 2.64 h ± 0.49 *** Unimanual movement time Stroke 1.85 h ± 0.68 vs. Controls 1.40 h ± 0.29 **** Composite movement time Stroke 2.66 h ± 0.73 vs. Controls 4.03 h ± 0.41 *** Movement time per region Below-MT Stroke UUL vs. AUL: 0.56 h ± 0.59 vs. 0.50 h ± 0.47 n.s. Controls DUL vs. NDUL: 1.64 h ± 0.55 vs. 1.60 h ± 0.79, n.s. Upper-T Stroke UUL vs. AUL: 1.58 h ± 0.56 vs. 0.34 h ± 0.21 *** Control DUL vs. NDUL: 1.82 h ± 0.60 vs. 1.20 h ± 0.60 *** Above-S Stroke UUL vs. AUL: 0.30 h ± 0.30 vs. 0.03 h ± 0.05 * Controls DUL vs. NDUL: 0.13 h ± 0.07 vs. 0.08 h ± 0.03 * Distance above shoulder Stroke UUL vs. AUL: 33.30 cm ± 11.52 vs. 18.76 cm ± 14.75 *** Control DUL vs. NDUL: 54.20 cm ± 9.32 vs. 48.80 cm ± 5.92 **
Bezuidenhout et al. [35]	Observational, Cross-sectional	3 triaxial ACC. (ActiGraph GT3Xþ) Both wrists and unaffected (Stroke) / dominant (HC) hip	Part 1: (HC, Stroke) Simulated ADL in a controlled environment. Part 2: (Stroke) Free ADL during waking hours for three consecutive days.	Stroke: N = 37 Age: 64.5 ± 11.7 y TAS: 3.0 ± 4.2 y (>=3 m) HC: N = 32 Age: 70.5 ± 10.4 y	MoCA ABILHand Katz ADL Index NIHSS CMSA	Vector Magnitude ratio (Wrists)	Part 1: Setting a table Stroke = 0.42 ± 0.32; HC = 0.95 ± 0.30 **** Washing dishes Stroke = 0.41 ± 0.37; HC = 1.12 ± 0.35 **** Walking Stroke = 1.13 ± 1.39; HC = 1.01 ± 0.21 n.s. Part 2: Sedentary Stroke 0.45 ± 0.61; HC = 0.88 ± 0.22 **** Active non-ambulation Stroke 0.54 ± 0.26; HC = 0.92 ± 0.11 **** Active ambulation Stroke 0.72 ± 0.31; HC = 1.00 ± 0.19 **** Correlation with VMR during free ADL (part 2): Sedentary periods CMSA r = 0.58 **** ABILHand r = 0.57 **** Active Non-Ambulation CMSA r = 0.57 **** ABILHand r = 0.63 **** Active Ambulation CMSA r = 0.49 *** ABILHand r = 0.43 **
Ann et al. [45]	Observational, Cross-sectional	2 IMU (Custom made) Only acc and gyroscope were used Both wrists	2 Parts: Part 1: 15 scripted activities Part 2: Free ADL during waking hours for 7 (ST) and 3 (HC) days.	Part 1: Stroke: N = 5 Age: 35.4 ± 13.21 y TAS: 45.8 ± 79.1 m (>=3 m) HC: N = 10 Age: 23.2 ± 3.21 y Part 2: Stroke: N = 5 Age: range 30–60 TAS: 2.0 ± 2.5 y (>=3 m) Mild-to-moderate impairment (FMA-UE) HC: N = 5 Age: not reported	FMA-UE] MAL, AAUT	Gross arm movements	Patients show reduced number of gross arm movement and some show asymmetrical values between arms (exact value not reported) Gross arm movements detect functional activities with 50–60% accuracy and eliminate non-functional activities with >90% accuracy but can’t identify functional activities involving fine finger movements and object stabilization. Some functional activities are identified as several smaller gross arm movements.
de Lucena et al. [36]	Observational, Cross-sectional (HC, Group 1), prospective (Group 2)	‘Manumeter’: custom equipment using ACC and magnetometers at the wrist and a magnetic ring to measure hand activity Affected wrist and index finger	Group 0 (HC): scripted hand and arm activities. Group 1: Clinical assessments, scripted activities and one day (walking hours) of free ADL. Group 2: Three times 1 day (walking hours) of free ADL. T1: first visit T2: T1 + 4 w T3: T1 + 4 m	Group 0 HC: N = 8 Age: 26.1 ± 3.0 SD Group 1 Stroke: N = 9 Age: 68 ± 9 y TAS: 30 ± 23 m Group 2 Stroke: N = 20 Age: 57 ± 15 y TAS: 40 ± 33	BBT, FMA-UE	‘HAND counts’ per hour: custom algorithm quantifying the amount of hand activity. Arm activity intensity	Scripted activities: Hand activities (50 movements): HC: 0.95 accuracy Group 1: ~0.8 accuracy Arm activities (200 arm movements): HC: error rate of 3.4% Sensor based measures during clinical assessment (group 1) Correlation between BTT score and: HAND counts per hour: r = 0.67 ** Arm activity intensity: r = 0.64 ** Correlation of FMA-UE score and: HAND counts per hour: r = 0.68 ** Arm activity intensity: r = 0.42 ** Sensor based measures during free ADL (group 1 and 2) Correlation between BBT score and HAND counts: r = 0.64 (statistical significance not reported)
Flury et al. [37]	Observational, Cross-sectional	6 IMUs Only acc. and gyroscope data were used. (Physilog^®^4, Gait Up Ltd., Lausanne, CH) Chest, both wrist, both shanks, impaired thigh.	Several hours of free ADL. (5.03 ± 1.1 h)	Stroke: N = 15 Age: 59.9 ± 9.8 y TAS: 6.5 ± 7.2 y (>3 m)	NIHSS FMA-UE MAL ARAT 10 MWT TUG BBS MRS Barthel Index	Activity time Number of steps Arm activity time Arm activity time ratio	Time walking (%): 12 ± 5.3 (2.9–20.2) Time standing (%): 19 ± 8.5 (8.7–33.2) Time sitting (%): 55 ± 13.5 (29.3–87.8) Time lying (%): 14 ± 15.6 (0.0–53.9) Sedentary time (sitting or lying, %): 69 Steps/hour (of recording time): 579 ± 243 (226–1066) Steps/walking episodes: 29 ± 13 (14–62) Longest walking episodes (in steps): 410 ± 277 (62–1148) AUL/UUL duration ratio during sitting (%): 74 ± 20 (52–124) AUL duration (in sec) during sitting normalized per hour (sec/hour): 866 ± 341 (326–1570)
Liao et al. [41]	Observational, Cross-sectional	Triaxial ACC (MicroMiniMotion logger, Ambulatory Monitoring). Both wrists	6 days of continuous recording (3 before, 3 after the intervention), except when in contact with large amounts of water	Stroke: Group 1: Robot assisted therapy N = 10 Age: 55.5 ± 11.1 y TAS: 33.4 ± 13.39 m Group 2: Control N = 10 Age: 54.56 ± 8.20 y TAS: 22.20 ± 17.47 m	FMA FIM MAL-AOU MAL-QOM ABILHand	Arm activity ratio	Arm activity ratio (pre / post intervention) change: Group 1: pre: 0.71 ± 0.99, post 0.76 ± 0.10 Group 2: pre: 0.69 ± 0.12, post 0.69 ± 0.11 Significant differences between group improvement values and clinical scores: FMA ***, MAL-AOU **, MAL-QOM ***, ABILHAND *, but n.s. with FIM.

Statistical significance levels: n.s. not significant; * *p* < 0.05; ** *p* < 0.01; *** *p* < 0.005; **** *p* < 0.001. Abbreviations in alphabetical order: AC = activity counts, ACC = accelerometer, ADL = Activities of Daily Living, AUL = affected upper limb, CIMT = Constraint Induced Movement Therapy, d = day(s), DUL = Dominant Upper Limb, Fall = Faller, h = hour(s), HC = Healthy controls, IMU = Inertial Measurement Unit, NDUL = Non Dominant Upper Limb, NFall = Non Faller, SRM = Standard Response Mean, ST = Stroke (patient), TAS = Time After Stroke, UL = Upper Limb, UUL = Unaffected Upper Limb, y = year(s), w = week(s). Clinical assessments in alphabetical order: 10 MWT = ten meters walk test, AAUT = Actual Amount of Use Test, ABILhand = measure of manual ability, ARAT = Action Research Arm Test, BBT = Box and Blocks Test, BBS = Berg Balance Scale, CMSA = Chedoke McMaster Stroke Assessment Scale, FIM (-M, -C) = Functional Independence Measure (Motor, Cognitive), FMA(-UE) = Fugl-Meyer Assessment (Upper Extremity), MAL(-AOU, -QOM) = Motor Activity Log (Amount of Activity, Quality of Movement), MRS = Modified Rankin Scale, NEADL = Nottingham Extended ADL, (SMS-)NIHSS = (Supplementary Motor Scale) National Institute of Health Stroke scale, SIS = Stroke Impact Scale, TL = Thumb Localization Test, TUG = Timed Up and Go test,.
